# Global Value Chain Participation and Green Innovation: Evidence from Chinese Listed Firms

**DOI:** 10.3390/ijerph19148403

**Published:** 2022-07-09

**Authors:** Shuang Meng, Huan Yan, Jiajie Yu

**Affiliations:** 1 School of International Trade and Economics, Central University of Finance and Economics, Beijing 100081, China; mengshuang@cufe.edu.cn; 2 Renmin Business School, Renmin University of China, Beijing 100872, China; yanhuan56@ruc.edu.cn; 3 Business School, Beijing Normal University, Beijing 100875, China

**Keywords:** global value chain, GVC participation, green innovation, developing countries, China

## Abstract

Green innovation is one of the most important approaches to prevent environmental pollution and foster sustainable development. Embedded in the global production networks, manufacturing firms have been found not only to be the main drivers of innovation but also the main polluters in developing countries. However, relatively few studies have systematically considered the effect of global value chain (GVC) participation on green innovation in the context of developing countries. By using a panel dataset of Chinese listed manufacturing firms, this study conducts panel data fixed-effect analyses and uses the instrumental variable two-stage least square model to investigate the effect of GVC participation on firms’ green innovation performance. The results show that increased GVC participation leads to improved green innovation performance of Chinese firms. Meanwhile, further heterogeneity analyses show that the impact of GVC participation on green innovation is more pronounced for firms with greater financial constraints, state-owned firms and firms in labor- or pollution-intensive industries, located in the eastern regions of China. Therefore, this study sheds light on the implication that actively participating in GVC is the key to promoting sustainable growth when facing the need for transformation in developing countries.

## 1. Introduction

With the acceleration of economic globalization and international trade liberalization, the manufacturing firms in developing countries have realized transformation and upgrading by participating in global value chain (GVC), which becomes an indispensable economic development paradigm. However, the longstanding conflict between economic development and environmental protection poses a challenge to sustainable development focusing on emission reduction and energy conservation. In recent years, Chinese manufacturing firms have not only contributed to rapid economic development but have also severely polluted the natural environment. Meanwhile, green innovation that integrates technological progress and green development is an important indicator that reflects sustainable development. In view of the fact that the environmental agenda has become a crucial and inevitable topic, whether GVC participation plays a role in affecting green innovation performance and environmental outcomes is worth investigating.

The emergence of GVC has considerably transformed and modernized the global manufacturing industry and international trade. Participation in GVC particularly helps firms in developing countries improve their productivities and competencies [1]. However, the issues of energy consumption and the environment, as well as GVC participation, have garnered increased attention both academically and practically in recent years. Evidence suggests that GVC has resulted in the transfer of carbon emissions and pollutant emissions from industrialized countries to developing countries [2]. There is an urgent need to resolve the conflict between economic development and environmental protection. Green innovation has recently attracted much attention, as it provides new technologies, goods, services and production processes that can help prevent environmental pollution and foster sustainable development [3]. In the context of China, manufacturing firms are not only the main drivers of innovation and development but also the main polluters during the development process. Therefore, green innovation within manufacturing firms facilitates the environmental improvement and industrial transformation of the economy.

There remains a research gap regarding how GVC participation affects enterprises’ green innovation performance, although there are several studies focusing on GVC participation and industrial upgrading or environmental issues. To address this research gap, we analyze a panel dataset of Chinese listed manufacturing firms from 2008 to 2014. Combining this with China’s customs database, we refer to the recent literature and calculate the GVC participation index for each firm [4]. The empirical analysis is then carried out using ordinary least squares and two-stage least squares (2SLS) estimations. Finally, our findings also highlight important heterogeneity in how GVC participation affects various types of firms’ green innovation.

The main contributions of this study are threefold. First, this paper sheds light on the relationship between GVC participation and green innovation in the context of developing countries. We seek to unpack the underlying mechanism through which the level of GVC participation affects green innovation performance. Specifically, GVC participation can introduce advanced green production technologies and increase the absorptive capacity of technology spillovers among various firms, which in turn boosts green innovation. Second, this study contributes to the existing literature by examining the environmental consequences of participating in GVC. Therefore, it provides new insights into the interrelationship between economic globalization, environmental protection and sustainable growth. Our findings reinforce the important role of GVC participation in shaping the environmental outcomes. In addition, we employ an instrumental variable (IV) approach to address potential endogeneity issues and establish a cause–effect relationship in the empirical study. Third, this study explores whether heterogeneity across firms, industries and regions influences the impacts of GVC participation on green innovation. The heterogeneity analysis allows policymakers to utilize targeted policies to enhance manufacturing firms’ capability of green innovation and sustainable development. It provides a useful tool for future research investigating longstanding conflicts between economic development and environmental protection, as well as the means to leverage green innovation and environmental performance in developing countries in the era of globalization.

The remainder of the paper is organized as follows. Section 2 provides the literature review. Section 3 describes the data and methodology, including data sources, measurements of variables, stylized facts and model specifications. Section 4 presents the results, including baseline findings and robustness checks. Section 5 shows the heterogeneity analysis based on firm, industry and region level. Section 6 illustrates the implications and limitations of the research. Finally, Section 7 concludes the paper.

## 2. Literature Review

Two strands of literature are related to this study: (a) GVC participation and industrial upgrading and (b) GVC participation and environmental issues in developing countries.

Previous research has demonstrated the significance of GVC participation in the economic growth and industrial upgrading of developing countries [5,6,7]. There is a growing consensus that the emergence of GVC represents a remarkable opportunity for promoting the ongoing transformations in developing countries [8,9]. GVC provides firms in developing countries with increased access to international markets, higher-quality inputs and technology transfer, which can give rise to research and development (R&D) spillovers, productivity improvements and growth [10,11]. Specifically, increased GVC participation allows firms to effectively and efficiently utilize the diverse knowledge they could acquire from their GVC partners, which can be considered as one of the most valuable resources in upgrading their technology and strengthening innovation capacity [12,13,14]. Meanwhile, inward-sourcing capability is critical for technical upgrade in GVC for emerging-economy firms [15]. It has been well documented in the literature that GVC participation boosts productivity and innovation efficiency [1], provides opportunities to modernize the industrial structure [16] and results in economic upgrading [17] for developing countries. Moreover, it is found that the business environment and foreign direct investment (FDI) significantly promote the status elevation on GVC, especially for labor-intensive industries [18]. The existing literature has suggested different channels through which GVC participation influences industrial upgrading and economic development of developing countries. The key underlying mechanisms include improved access to global markets, increased availability and quality of imported inputs, advanced technology and knowledge transfers and enhanced innovation capacity. Nevertheless, all of these major transmission channels are essentially theoretical, and the materialization of the above-mentioned theoretical effects is still uncertain. Hence, empirical studies are of vital importance to test the theoretical predictions in the context of developing countries. In addition, investigating the relationship between GVC participation and industrial upgrading in China could provide lessons for other developing countries to foster economic development in this age of globalization.

Another strand of the literature has focused on the environmental and energy consequences of GVC [19,20,21]. Two opposing hypotheses emerged with the advancement of globalization. The first “pollution haven hypothesis” states that developing countries increase pollution emissions when embedded in GVC. In particular, previous studies provided supportive evidence for this hypothesis [2,22]. The other “pollution halo hypothesis” indicates that developing countries may reduce pollution emissions when embedded in GVC because industrialized countries may diffuse their advanced technology and experience into developing countries. GVC participation has been found to reduce carbon emissions and environmental pollution in developing countries [23,24]. Meanwhile, it has been demonstrated that there is an inverted U-shaped relationship between GVC participation and energy intensity [25]. To summarize, there is still no clear consensus in the literature regarding the relationship between GVC participation and environmental outcomes in developing countries. It appears that the existing studies have three major shortcomings: (1) they have mostly relied on GVC participation indicators at the aggregate country or industry level, as opposed to detailed measures of GVC participation at the disaggregate firm level, which may result in measurement errors; (2) they have not systematically addressed the potential endogeneity problem, which could suffer from biases due to reverse causality; and (3) they have not fully considered the heterogeneity across firms, industries and regions, which might ignore the important heterogeneous effects of GVC participation in developing countries. As the largest emerging economy, China has actively participated in GVC through forward and backward linkages. Thus, whether Chinese firms’ GVC participation plays a role in shaping the environmental outcomes remains to be further studied.

Green innovation has not yet been adequately investigated as a critical bridge to resolve conflicts between economic expansion and environmental protection. Although the economic and environmental effects of GVC participation in developing countries have been extensively studied in the existing literature, relatively scarce research has systematically examined the effect of GVC participation on green innovation. To fill this critical gap, this study aimed to explore the impact of GVC participation on firms’ green innovation performance using a panel dataset of listed Chinese manufacturing firms, which sheds empirical light on the role of GVC participation on green innovation in the largest developing country.

In the era of globalization, green innovation performance typically hinges on the import and export of intermediate goods, and the implication of GVC participation is the share of foreign value added in total exports [4]. With the gradual deepening of the “new development philosophy” in China, featuring innovative, coordinated, green, open and shared growth, Chinese manufacturing firms are at the critical moment of transformation from extensive development to intensive development. Increasing attention has been paid to green innovation performance of Chinese manufacturing firms, which could consider both economic development and environmental protection. Specifically, GVC participation can bring about advanced green production technologies and enhance the absorptive capacity of technology spillovers, which in turn promotes green innovation. Therefore, the objective of this study is to examine the influencing mechanism of GVC participation on Chinese manufacturing firms’ green innovation performance.

## 3. Data and Methodology

### 3.1. Data and Sample

To investigate the relationship between manufacturing firms’ GVC participation and green innovation performance, we merge two databases: (1) firm-level green innovation data and (2) firm-product level trade data.

The first database is the listed firms’ database sourced from the China Stock Market Accounting Research (CSMAR) database. It provides detailed information of each listed firm’s financial, operating and corporate governance information, among others. This database has been widely used in previous studies regarding Chinese listed firms’ green innovation performance [26,27].

We also use international trade data from China’s Customs Database, which covers Chinese exporters and importers and provides detailed information on the firm–product–country–trade mode level transaction, including trade values, quantities, units, product codes (i.e., 8-digit harmonized system (HS)), importing or exporting countries, contact information, firm ownership type and trade mode. This is by far the most detailed international trade database in China, which enables us to calculate the GVC participation index [4,10].

The focus of this study is Chinese listed manufacturing firms, and all indicators are constructed at the firm level. To calculate firm-level GVC participation, we match these two databases by their contact information (i.e., firm names and contact details). After removing observations with missing data and winsorizing extreme values at the 1% level, we obtain an unbalanced dataset with 4577 firm–year observations from 2008 to 2014 as the final sample.

### 3.2. Variables

#### 3.2.1. Dependent Variable

The dependent variable is green innovation performance within firms (*GI*). Green innovation reflects the comprehensive ecological, economic and social development of the firm. It is beneficial for enhancing the competitiveness of the firm, thus promoting the upgrading of industrial structures and high-quality economic growth [28]. Following previous studies [29,30,31], it is calculated by the total number of green invented patent applications, which reflects the firm’s dedication and effort in terms of green innovation for that year.

#### 3.2.2. Independent Variable

The independent variable of this study is GVC participation of the firm (*GVC*). A wide variety of approaches to GVC participation calculation rely on World Input-Output Database, which are based on the industry level, and they tend to ignore the heterogeneity of firms. Following previous studies [4,32], *GVC* is measured by the proportion of foreign value added in total export in this study. As shown in the Equation (1), $V_{F}$ and EX represent the firms’ export foreign value added and export value, respectively. The customs dataset is employed to calculate export trade value (*EX*) and import trade value (*IM*), whereas domestic sales (*D*) are calculated by subtracting income from export value.

(1) $$GVC_{f}=\frac{V_{F}}{EX}=\frac{IM_{Ap}+EX_{o}\left[IM_{Ao}/\left(D+EX_{o}\right)\right]}{EX}$$

where the subscripts *o* and *p* denote the ordinary trade and the processing trade, respectively. We assume that processing imports consist of foreign value added, and ordinary imports consist of intermediate goods and final goods, where the former is used only for foreign value added. We classify product categories into intermediate inputs, capital goods and consumer goods, as suggested by the relevant literature [4]. Intermediate inputs of ordinary imports are treated as an input for producing foreign value added. The numerator of the right-hand side consists of value added created by processing trade (*IM_Ap_*), which is measured by inputs via processing import; foreign value added created by processing trade ($EX_{o}\left[IM_{Ao}/\left(D+EX_{o}\right)\right])$, which assumes that the intermediate input via ordinary trade is used in proportion to domestic sales and exports.

#### 3.2.3. Control Variables

Following previous studies [26,27], we control a comprehensive set of variables that may affect the green innovation performance.

1. Income (*Revenue*), measured by the natural logarithm of operating revenue in the year.
2. Firm age (*Age*), measured by the natural logarithm of firm age.
3. Government subsidy (*Subsidy)*, measured by the natural logarithm of government subsidy.
4. Firm size (*Asset*), measured by the natural logarithm of the total net asset.
5. Firm financial development potential (*Tobinq*), measured by the ratio of firm market value to capital replacement cost.
6. Industry competition *(HHI)*, measured by the Herfindahl–Hirschman index within the industry.

### 3.3. Stylized Facts

The descriptive statistics of all the variables are shown in Table 1. As indicated therein, the mean of green innovation is 1.5045, which implies that the current green innovation of China’s listed firms needs to be improved. Moreover, all the other variables have large variations over time, which enables us to exploit these variations and conduct the regression analysis.

**Figure 1:**
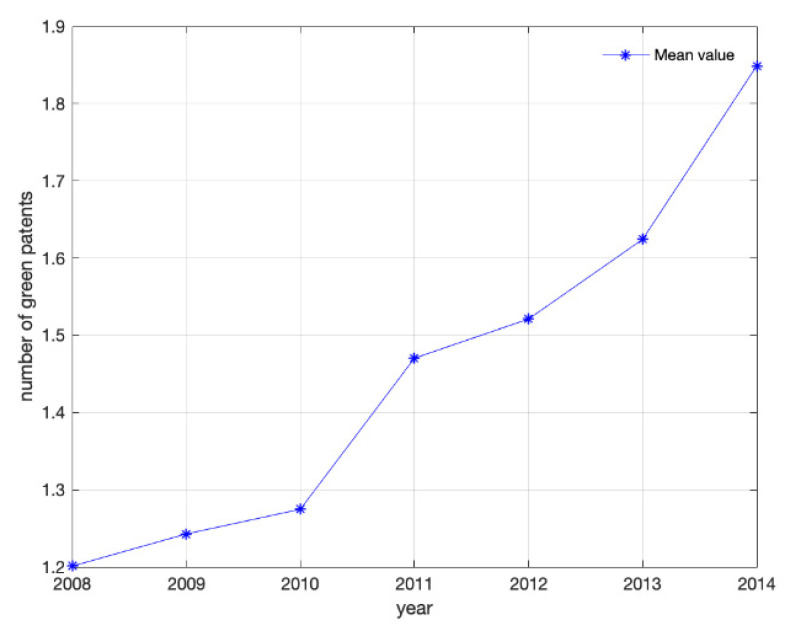
Green innovation performance trend (2008 to 2014).

Furthermore, Figure 1 plots the mean of green invention of listed Chinese firms from 2008 to 2014. It shows that green innovation grew from 1.2011 in 2008 to 1.8488 in 2014, which reflects that firms are paying considerable attention to green development. During this period, the Chinese government also promulgated a series of policy measures to promote green growth. For example, in the 12th Five-Year Plan of 2011, it was proposed to focus on energy conservation and emission reduction to promote green and low-carbon development.

### 3.4. Empirical Model Specification

To test the relationship between GVC participation and green innovation performance, we estimate the following specification:

(2) $$GI_{ft}=\beta_{0}+\beta_{1}GVC_{ft}+X_{ft}^{’}\gamma +F_{f}+F_{i}+F_{t}+\epsilon_{ft}$$

where *f* represents the firm, *t* denotes the year, and *i* characterizes industry. The dependent variable *GI* denotes the green innovation, and the independent variable *GVC* represents the degree of firm participation in GVC. $X_{ft}$ is a set of control variables that may affect green innovation. γ is a vector of estimated coefficients of control variables. $F_{f}$, $F_{i}$ and $F_{t}$ denote the firm, industry and year fixed effects, respectively, which are included to control for unobservable characteristics at firm, industry and year levels. Finally, $\epsilon_{ft}$ represents a random error term. For all the estimates, robust standard errors are reported.

## 4. Results

### 4.1. Baseline Regression Results

The baseline results are presented in Columns 1 and 2 of Table 2. As shown in Column 1, the regression coefficient of GVC participation is 0.2537 and statistically significant at the 5% level, which indicates that GVC participation has a positive effect on the firms’ green innovation performance. In Column 2, we add other control variables, and the coefficient of GVC participation remains positive and significant. Among the control variables, the estimated coefficients of revenue (*Revenue*) and size (*Asset*) are positive and significant, suggesting that these factors would promote green innovation performance. One possible reason is that green innovation activities usually require huge capital investment; thus, large firms have sufficient financial ability to carry out green research and development.

### 4.2. Robustness Checks

#### 4.2.1. Alternative Sample

The 2008 financial crisis was an important event that affected the world economy, and it inevitably had a damaging effect on the development of firms participating in the GVC. Considering this circumstance, this study excluded the sample of 2008 and the adjacent year 2009 and only retained the sample from 2010 to 2014 to re-estimate the baseline model. According to the results shown in Column 3 of Table 2, the coefficient of *GVC* remains positive and significant at the 5% level, which is qualitatively consistent with the baseline result.

#### 4.2.2. Endogeneity

The endogeneity concern occurs when GVC activities may be influenced by green innovation, or both of these could be jointly affected by other factors. To solve this problem, we refer to the literature [33] and utilize the cube of the difference between a firm’s GVC participation and its mean value within the same industry or the same province as instrumental variables. Moreover, these two instrumental variables are used to test the robustness of the baseline regression using the 2SLS method. As reported in Table 3, Columns 1 and 2 show the results of the cube of the difference between a firm’s GVC participation and its mean value within the same industry as the instrumental variable (*IV1*), and Columns 3 and 4 represent the cube of the same difference value within the same province as the other instrumental variable (*IV2*). The *F*-value of the instrument variable in the first stage is greater than 10, which reflects that the instruments are not weak. Both instrumental variables are highly correlated with *GVC*, which indicates the relativeness of the instrumental variables. After accounting for the endogeneity, the coefficients of *GVC* are still significantly positive at 5%, demonstrating the robustness of the baseline findings.

## 5. Further Analysis: Heterogeneity Analysis

### 5.1. Firm Heterogeneity

The existing literature confirmed that different types of firms have different financial conditions and institutional logics [26,34], which may heterogeneously affect green innovation. Thus, the firm heterogeneity analysis is reported in this subsection.

#### 5.1.1. Firm Heterogeneity by Financial Constraints

Since green innovation is associated with a substantial investment, it requires the firms to have good financial conditions. To investigate the consequences of financial condition heterogeneity, we refer to a relevant study [26] and divide the sample into two groups: high and low financial constraints. When calculating financial constraints, we refer to previous literature to calculate the size–age (SA) index [35]. A higher SA index value means that the firm is more financially constrained. Then, a firm is placed in the high financial constraints group if the SA index is larger than the median value; otherwise, it is treated as belonging to the low financial constraints group. As shown in Table 4, the regression coefficient of GVC participation in the high financial constraints group is positive and statistically significant. It indicates that GVC participation has a positive effect on green innovation only for firms with high financial constraints.

#### 5.1.2. Firm Heterogeneity by Ownership

As the major economic players, state-owned enterprises play an important role in the economic development of emerging markets [34]. In particular, there are differences in government regulation between state-owned and non-state-owned enterprises. Therefore, this study classifies the sample into state-owned enterprises and non-state-owned enterprises according to their ownership forms. As reported in Table 5, the coefficients of *GVC* in the two groups are both positive and statistically significant, although the coefficient in stated-owned firms is higher. It indicates that compared with non-state-owned enterprises, state-owned enterprises participating in GVC may promote green innovation more strongly.

### 5.2. Industry Heterogeneity

Firms in different industries may experience different energy utilization efficiencies, production environments, etc.; thus, it is necessary to investigate the heterogeneity across industries.

#### 5.2.1. Industry Heterogeneity by Factor Intensity

Firstly, we classify the sample according to their factor intensity, i.e., the labor-intensive industry, capital-intensive industry and technology-intensive industry [36]. The classification of the industries is presented in the Appendix A, Table A1. As shown in Table 6, the coefficient of GVC participation in the labor-intensive industry sample is 0.4344 and statistically significant at the 5% level. Meanwhile, the coefficients of capital-intensive and technology-intensive industries are insignificant. These results show that only GVC participation by firms in the labor-intensive industry can promote green innovation.

#### 5.2.2. Industry Heterogeneity by Pollution Intensity

Relying on first-mover advantages, the developed countries at the high end of GVC transfer the processing and assembly sectors to developing countries, and developing countries are forced to take over these industries with high pollution and high energy consumption [37]. Thus, it is necessary to explore the heterogeneous effects across industries with different pollution intensities. Following previous research [38], we divide the sample into pollution-intensive industries and non-pollution-intensive industries. The pollution-intensive industries consist of eight “severe pollution” industries, such as textile, paper and paper products manufacturing. The classification of the industries is presented in the Appendix A, Table A1. Table 7 shows the estimation results, and the coefficients are 0.3477 and 0.2119 for pollution-intensive and non-pollution-intensive industries, respectively. Both coefficients are significantly positive, and the coefficient of GVC in the pollution-intensive industry is positive at the 5% level and larger than that in non-pollution-intensive industries. It reflects that companies in the pollution-intensive industry participating in GVC have a greater positive impact on green innovation.

### 5.3. Regional Heterogeneity

The geographical imbalance is a significant characteristic of China’s economic development [39], where the eastern region is more economically developed. Benefiting from superior geographical location, the eastern region reformed and opened earlier, resulting in the establishment of a large number of firms. Therefore, we divide listed firms into eastern, central, western and northeastern regions according to their geographic locations. According to the results in Table 8, only the coefficient of GVC participation in the eastern region is positive and significant. It shows that the effect of GVC participation on green innovation performance is unbalanced.

## 6. Discussion

### 6.1. Empirical Results Discussion

This study takes Chinese listed manufacturing firms from 2008 to 2014 as final sample to analyze the effect of participating in GVC on green innovation. The baseline result and robust test support the hypothesis and reveal that intense participation in GVC is conducive to green innovation. International trade, FDI and outward FDI are the key means of GVC participation for Chinese firms [40]. When firms participate in GVC, they would not only import advanced intermediate products but also learn advanced management experience from multinational enterprises [18,41]. In addition, it facilitates the transmission of sophisticated technology and management experience across regions and nations, thereby promoting green innovation. Moreover, other studies have found that developing countries, such as China, could improve their innovation competitiveness related to high value added from the process of production fragmentation [42].

In addition, considering China’s economic development characteristic, we conduct heterogeneity analysis at the firm, industry and region levels.

In the firm heterogeneity analysis, the analysis based on classification by financial constraints provides an interesting finding: participation in GVC by firms with high financial constraints has a positive effect on green innovation. A possible explanation could be that firms with high financial constraints are inclined to participate in GVC and build up cooperative relationships with international trading partners [43]. GVC participation could help firms broaden capital sources, which could mitigate the negative effects of financial constraints on green innovation. In addition, the group regressions by ownership demonstrate that compared with non-state-owned enterprises, the promotion effect of participating in GVC on green innovation is more pronounced in state-owned enterprises. This could be due to the fact that state-owned enterprises are part of China’s political system and have closer ties to the government [44]. On the one hand, state-owned enterprises may face strict state governance [34]; thus, their managers have a stronger green awareness. Previous study also confirmed that institutional pressure is an important driving factor for green innovation [45]. On the other hand, many environment-related projects promoted by the state government usually require green technologies, and if enterprises have obtained green patents, it would be conducive to cooperate with the government [44].

In the industry heterogeneity analysis, the regression results by factor intensity reveal that only the coefficient of GVC participation in labor-intensive industry is positive and significant. Labor-intensive industries include processing of food from agricultural products, manufacture of textiles, etc., and China has accumulated ample production and management experience in these industries. Compared with other industries, labor-intensive industries have relatively lower technical barriers. Therefore, firms are more likely to promote green innovation through learning effects and technology spillovers in the GVC network. In contrast, firms engaging in capital-intensive and technology-intensive activities may face low-end locking by industrialized countries, such that they are limited in absorbing and upgrading technology [46,47], which may impede green innovation. The heterogeneous analysis across industries with different pollution intensities shows that the positive effect of GVC participation on green innovation is more pronounced in the pollution-intensive industry. This could be explained by the fact that firms in these pollution-intensive industries have stronger motivation to promote green growth to break through resource limitation and environmental constraints [38].

In the region heterogeneity analysis, the coefficient of GVC participation in the eastern region is positive and significant. A possible explanation could be that large numbers of firms are concentrated in the eastern region, and the communication among firms is more frequent [48], which promotes the spillover effect through information sharing and is ultimately conducive for green innovation.

### 6.2. Implications

Green innovation has attracted the attention of scholars and practitioners, in that it is one of the most important means for firms to manage the environmental concerns and achieve sustainability. The manufacturing sector plays a significant role in economic development and environmental protection. Manufacturing firms in developing countries are also the main participants in GVC activities. Thus, it is critical to understand the effect of GVC participation on green innovation performance.

These findings have important theoretical and policy implications for researchers, practitioners and policymakers. First, this study systematically investigates the impact of GVC participation on green innovation in China. Our paper provides supportive evidence for the research hypothesis that GVC participation has a positive effect on Chinese manufacturing firms’ green innovation performance, which is in accordance with the theoretical framework developed in the recent literature [40,41,42]. This study not only expands research on resolving conflicts between economic expansion and environmental protection but also provides theoretical support for Chinese manufacturing firms to participate actively in GVC. It contributes to a better understanding of utilizing GVC participation to improve green innovation in developing countries. Second, this study demonstrates that, in practice, critical knowledge, important skills and advanced technologies related to the process of green innovation flow across countries through GVC participation. Hence, economic globalization plays a significant role in facilitating the diffusion of green technology and elevating the performance of green innovation. It sheds light on how integrating into the process of economic globalization promotes environmental performance and sustainable growth for developing countries. Third, this study reveals the external and internal barriers to green innovation by scrutinizing the level of GVC participation and firm characteristics. It enables the managers of manufacturing firms to better understand and cope with the potential barriers to the green innovation they want to practice, which will enhance the firm’s capability of sustainable development in the era of globalization. Finally, this study explores the heterogeneous effects stemming from GVC participation across firms, industries and regions. Policymakers in developing countries could utilize targeted policies to incentivize firms, industries and regions to acquire advanced knowledge and technologies, thereby creating a favorable environment in which to transfer green technology and improve green innovation performance.

### 6.3. Limitations and Future Research Directions

Although this study makes several contributions to the literature, there are still some limitations to be addressed in future research. First, our sample is based on China, one of the largest developing countries in the world, but it would be conducive to investigate whether our findings are generalizable to manufacturers from other developing countries in Asia, while accounting for heterogeneity and complexity across countries. Future research can study the positive externalities and mechanisms of GVC activities on green innovation in more developing countries. It contributes to realizing economic transformation in developing countries and provides more empirical experience for relevant theories in development economics and environmental economics. Second, due to data limitation, our research sample is restricted to Chinese listed firms. However, small and medium enterprises are also active participants in economic development and globalization. It can also be a fruitful direction for future research to determine the differences between listed firms and those small and medium enterprises in terms of GVC participation and green innovation performance with more in-depth survey data. Third, this study is based on firm-level data, and we are unable to observe how firms organize internal resources to drive green innovation. In future research, we can employ qualitative methodology to conduct field research in specific companies. Combined with corporate management theory and organization theory, it is valuable to provide qualitative insights into the relationship between GVC participation and green innovation. Finally, as the digital economy has developed rapidly around the world, the organization of GVC and its mechanisms on manufacturing firms may have changed. It would be interesting to investigate the relationship between GVC participation and green innovation in the context of the digital era with more recent data.

## 7. Conclusions

This study investigates the effect of GVC participation on green innovation performance. Using a panel dataset of Chinese listed manufacturing firms from 2008 to 2014, we conduct panel data analyses, 2SLS estimation and heterogeneity analyses and test the mechanisms. The results show that GVC participation increases green innovation, with the positive effect being more pronounced for firms with greater financial constraints, state ownership, in labor-intensive industries, in pollution-intensive industries and in the eastern regions of China. These findings are attributed to the specific characteristics of the Chinese context. In sum, this study demonstrates that actively participating in GVC is the key to promoting sustainable growth when facing the need for transformation in developing countries.

## Figures and Tables

**Table 1:** Descriptive statistics.

Variables	Definition	Observation	Mean	S.D.
*GI*	Green innovation	4577	1.5045	1.8194
*GVC*	The degree of participation in GVC (%)	4577	0.2108	0.3514
*Revenue*	Total operating revenue (log)	4577	21.0180	1.2235
*Age*	Firm age (log)	4577	1.5802	0.8466
*Subsidy*	Government subsidy (log)	4577	15.7069	2.2755
*Asset*	Total net asset (log)	4577	21.5395	1.0118
*Tobinq*	The ratio of market value to capital replacement cost	4577	2.0522	1.4834
*HHI*	The degree of competition in the industry (HHI index)	4577	0.2696	0.1073

**Table 2:** Baseline regression and robust checks I.

	Baseline Regression		Alternative Sample (2010–2014)
	(1)	(2)	(3)
*GVC*	0.2537 ** (0.0988)	0.2483 ** (0.0985)	0.2565 ** (0.1170)
*Revenue*		0.1982 ** (0.0886)	0.1583 (0.1056)
*Age*		−0.1970 *** (0.0725)	−0.1622 * (0.0856)
*Subsidy*		−0.0007 (0.0112)	0.0139 (0.0155)
*Asset*		0.2718 ** (0.1080)	0.2359 * (0.1360)
*Tobinq*		0.0115 (0.0235)	0.0058 (0.0263)
*HHI*		−0.0820 (0.9457)	−0.8258 (1.4204)
*Constant*	1.4510 *** (0.0265)	−8.2474 *** (1.7748)	−6.6658 *** (2.2254)
Firm FE	Yes	Yes	Yes
Industry FE	Yes	Yes	Yes
Year FE	Yes	Yes	Yes
Observations	4577	4577	3793

Note: Robust standard errors in parentheses. * *p* < 0.10, ** *p* < 0.05, *** *p* < 0.01.

**Table 3:** Robustness checks II: 2SLS estimation.

	(1)	(2)	(3)	(4)
	First-Stage	Second-Stage	First-Stage	Second-Stage
Dependent Variable	*GVC*	*GI*	*GVC*	*GI*
*IV1*	1.5587 ***			
	(0.0193)			
*IV2*			1.7864 ***	
			(0.0198)	
*GVC*		0.6018 **		0.6419 **
		(0.2388)		(0.2547)
*Revenue*	0.0112	0.2005 **	0.0049	0.1984 **
	(0.0089)	(0.0886)	(0.0083)	(0.0886)
*Age*	0.0026	−0.1944 ***	0.0001	−0.1957 ***
	(0.0073)	(0.0725)	(0.0068)	(0.0725)
*Subsidy*	0.0006	−0.0007	−0.0003	−0.0010
	(0.0011)	(0.0112)	(0.0010)	(0.0112)
*Asset*	−0.0059	0.2721 **	−0.0099	0.2703 **
	(0.0109)	(0.1080)	(0.0101)	(0.1080)
*Tobinq*	−0.0020	0.0122	−0.0029	0.0116
	(0.0024)	(0.0235)	(0.0022)	(0.0235)
*HHI*	−0.0643	−0.0798	−0.1052	−0.0980
	(0.0953)	(0.9461)	(0.0887)	(0.9460)
Firm FE	Yes	Yes	Yes	Yes
Industry FE	Yes	Yes	Yes	Yes
Year FE	Yes	Yes	Yes	Yes
F-value	6525.061		8099.532	
Observations	4577	4577	4577	4577

Note: Robust standard errors in parentheses. * *p* < 0.10, ** *p* < 0.05, *** *p* < 0.01.

**Table 4:** Firm heterogeneity analysis: Financial constraints.

	Financial Constraints	
	(1)	(2)
	High Financing Constraints	Low Financing Constraints
*GVC*	0.3703 ** (0.1685)	0.0986 (0.1014)
*Revenue*	0.2199 (0.1466)	0.2003 ** (0.0956)
*Age*	−0.1405 (0.1282)	−0.0989 (0.0762)
*Subsidy*	−0.0112 (0.0205)	0.0089 (0.0108)
*Asset*	0.2173 (0.1756)	−0.0283 (0.1291)
*Tobinq*	0.0757 * (0.0419)	−0.0354 (0.0241)
*HHI*	−0.6417 (1.4559)	1.1867 (1.1296)
Constant	−7.4691 ** (2.9439)	−2.4785 (2.1788)
Firm FE	Yes	Yes
Industry FE	Yes	Yes
Year FE	Yes	Yes
Observations	2234	2343

Note: Robust standard errors in parentheses. * *p* < 0.10, ** *p* < 0.05, *** *p* < 0.01.

**Table 5:** Firm heterogeneity analysis: Ownership.

	Ownership	
	(1)	(2)
	State-Owned	Non-State-Owned
*GVC*	0.3159 * (0.1885)	0.1881 * (0.1139)
*Revenue*	0.3993 ** (0.1633)	0.1197 (0.1055)
*Age*	−0.1950 (0.2017)	0.0179 (0.0859)
*Subsidy*	−0.0177 (0.0193)	0.0144 (0.0138)
*Asset*	0.3046 (0.1909)	0.2631 ** (0.1312)
*Tobinq*	0.0259 (0.0516)	0.0042 (0.0257)
*HHI*	−0.8491 (2.3330)	−0.3103 (0.9923)
Constant	−12.8771 *** (3.4091)	−6.8631 *** (2.0887)
Firm FE	Yes	Yes
Industry FE	Yes	Yes
Year FE	Yes	Yes
Observations	1445	3132

Note: Robust standard errors are in parentheses. * *p* < 0.10, ** *p* < 0.05, *** *p* < 0.01.

**Table 6:** Industry heterogeneity analysis: Factor intensity.

	Factor Intensity		
	(1)	(2)	(3)
	Labor Intensive	Capital Intensive	Technology Intensive
*GVC*	0.4344 ** (0.1823)	0.1832 (0.1331)	0.2535 (0.1603)
*Revenue*	−0.1155 (0.2586)	0.1725 (0.1304)	0.2409 * (0.1316)
*Age*	−0.1628 (0.1560)	−0.2571 *** (0.0979)	−0.1961 * (0.1172)
*Subsidy*	−0.0119 (0.0160)	0.0017 (0.0127)	0.0037 (0.0226)
*Asset*	0.2600 (0.2810)	0.1622 (0.1528)	0.2497 (0.1685)
*Tobinq*	0.0041 (0.0538)	−0.0549 (0.0365)	0.0311 (0.0347)
*HHI*	0.8204 (0.8787)	1.3642 (1.4384)	−4.2010 ** (2.0721)
*Constant*	−1.9801 (4.8908)	−5.7771 ** (2.6480)	−7.4597 *** (2.7472)
Firm FE	Yes	Yes	Yes
Industry FE	Yes	Yes	Yes
Year FE	Yes	Yes	Yes
Observations	339	1839	2399

Note: Robust standard errors are in parentheses. * *p* < 0.10, ** *p* < 0.05, *** *p* < 0.01.

**Table 7:** Industry heterogeneity analysis: Pollution intensity.

	Pollution Intensity	
	(1)	(2)
	Pollution-Intensive	Non-Pollution-Intensive
*GVC*	0.3477 ** (0.1422)	0.2119 * (0.1274)
*Revenue*	0.2008 (0.1670)	0.2098 ** (0.1065)
*Age*	−0.3670 *** (0.1219)	−0.1740 * (0.0906)
*Subsidy*	−0.0031 (0.0138)	−0.0002 (0.0159)
*Asset*	0.1117 (0.1764)	0.3157 ** (0.1358)
*Tobinq*	−0.0500 (0.0466)	0.0189 (0.0278)
*HHI*	3.4928 * (2.0979)	−0.4559 (1.1081)
*Constant*	−5.5977 * (3.2024)	−9.2773 *** (2.1774)
Firm FE	Yes	Yes
Industry FE	Yes	Yes
Year FE	Yes	Yes
Observations	1255	3322

Note: Robust standard errors are in parentheses. * *p* < 0.10, ** *p* < 0.05, *** *p* < 0.01.

**Table 8:** Regional heterogeneity analysis.

	Region Heterogeneity			
	(1)	(2)	(3)	(4)
	Eastern	Middle	West	Northeast
*GVC*	0.3501 *** (0.1247)	0.0561 (0.2327)	0.1140 (0.3386)	0.0336 (0.1898)
*Revenue*	0.1400 (0.1158)	0.2440 (0.2309)	0.2074 (0.2361)	0.3596 * (0.2111)
*Age*	−0.2171 ** (0.0883)	−0.2985 * (0.1788)	0.0385 (0.2929)	−0.1007 (0.1933)
*Subsidy*	0.0051 (0.0142)	0.0133 (0.0286)	−0.0195 (0.0415)	−0.0139 (0.0182)
*Asset*	0.2988 ** (0.1365)	0.3862 (0.2663)	0.1121 (0.3522)	0.2981 (0.2717)
*Tobinq*	0.0033 (0.0279)	0.0310 (0.0569)	0.0097 (0.1071)	0.1150 * (0.0636)
*HHI*	0.3719 (1.1460)	−1.7673 (2.0652)	1.0734 (3.8960)	−6.2563 ** (3.1153)
Constant	−7.7794 *** (2.1519)	−11.3319 ** (4.6862)	−5.3627 (7.5537)	−10.8629 *** (3.5076)
Firm FE	Yes	Yes	Yes	Yes
Industry FE	Yes	Yes	Yes	Yes
Year FE	Yes	Yes	Yes	Yes
Observations	3253	676	470	178

Note: Robust standard errors are in parentheses. * *p* < 0.10, ** *p* < 0.05, *** *p* < 0.01.

## Data Availability

Data available in the chargeable databases China Security Market and Accounting Research (CSMAR) database and China Customs Database.

## Abbreviations

GVC	Global value chain
GI	Green innovation
2SLS	Two-stage least squares
IV	Instrumental variable
R&D	Research and development
FDI	Foreign direct investment
CSMAR	China Stock Market Accounting Research
HS	Harmonized system
HHI	Herfindahl–Hirschman Index
SA	Size–Age

## Appendix A

**Table A1:** Industry classification.

Code	Industry Name	Factor Intensity	Pollution- Intense Industry
C13	Processing of food from agricultural products	Labor	Yes
C14	Manufacture of foods	Capital	No
C15	Manufacture of wine, drinks and refined tea	Capital	No
C17	Manufacture of textiles	Labor	Yes
C18	Manufacture of textile wears and apparel	Labor	No
C19	Manufacture of leather, fur, feather and related products and footwear	Labor	No
C20	Processing of timber, manufacture of wood, bamboo, rattan, palm and straw products	Labor	No
C21	Manufacture of furniture	Labor	No
C22	Manufacture of paper and paper products	Capital	Yes
C23	Printing, reproduction of recorded media	Capital	No
C24	Manufacture of artwork and articles for culture, education, sports and recreation	Capital	No
C25	Processing of petroleum, coking, processing of nuclear fuel	Capital	Yes
C26	Manufacture of raw chemical material and chemical products	Capital	Yes
C27	Manufacture of medicines	Technology	No
C28	Manufacture of chemical fibers	Technology	No
C29	Manufacture of rubber and plastic	Capital	No
C30	Manufacture of non-metallic mineral products	Capital	Yes
C31	Smelting and pressing of ferrous metals	Capital	Yes
C32	Smelting and pressing of non-ferrous metals	Capital	Yes
C33	Manufacture of metals products	Capital	No
C34	Manufacture of general-purpose machinery	Capital	No
C35	Manufacture of special-purpose machinery	Technology	No
C36	Manufacture of automobiles	Technology	No
C37	Manufacture of railway, ship, aerospace and other transport equipment	Technology	No
C38	Manufacture of electrical machinery and equipment	Technology	No
C39	Manufacture of computer, communications and other electronic equipment	Technology	No
C40	Manufacture of measuring instruments	Technology	No
C41	Other manufactures	Technology	No
C42	Utilization of waste resources	Capital	No
C43	Repairs services of metal products, machinery and equipment	Capital	No
